# Oil degradation and biosurfactant production by the deep sea bacterium *Dietzia maris* As-13-3

**DOI:** 10.3389/fmicb.2014.00711

**Published:** 2014-12-16

**Authors:** Wanpeng Wang, Bobo Cai, Zongze Shao

**Affiliations:** ^1^Key Laboratory of Marine Genetic Resources, Third Institute of Oceanography, State Oceanic AdministrationXiamen, China; ^2^State Key Laboratory Breeding Base of Marine Genetic Resources, Third Institute of Oceanography, State Oceanic AdministrationXiamen, China; ^3^Collaborative Innovation Center of Deep Sea Biology, Third Institute of Oceanography, State Oceanic AdministrationXiamen, China; ^4^Key Laboratory of Marine Genetic Resources of Fujian ProvinceXiamen, China; ^5^Fujian Collaborative Innovation Center for Exploitation and Utilization of Marine Biological ResourcesXiamen, China; ^6^Life Science College, Xiamen UniversityXiamen, China

**Keywords:** *Dietzia*, biosurfactant, di-rhamnolipid, hydrocarbon degradation, genome sequence, biosynthesis pathway

## Abstract

Recent investigations of extreme environments have revealed numerous bioactive natural products. However, biosurfactant-producing strains from deep sea extreme environment are largely unknown. Here, we show that *Dietzia maris* As-13-3 isolated from deep sea hydrothermal field could produce di-rhamnolipid as biosurfactant. The critical micelle concentration (CMC) of the purified di-rhamnolipid was determined to be 120 mgL^−1^, and it lowered the surface tension of water from 74 ± 0.2 to 38 ± 0.2 mN m^−1^. Further, the alkane metabolic pathway-related genes and di-rhamnolipid biosynthesis-related genes were also analyzed by the sequencing genome of *D. maris* As-13-3 and quantitative real-time PCR (Q-PCR), respectively. Q-PCR analysis showed that all these genes were induced by *n*-Tetradecane, *n*-Hexadecane, and pristane. To the best of our knowledge, this is first report about the complete pathway of the di-rhamnolipid synthesis process in the genus *Dietzia*. Thus, our study provided the insights into *Dietzia* in respects of oil degradation and biosurfactant production, and will help to evaluate the potential of *Dietzia* in marine oil removal.

## Introduction

Biosurfactants (BS) are surface activity compounds possessing both hydrophilic and hydrophobic moieties (Satpute et al., 2010). Due to the diversity of microorganisms and their metabolites, microorganisms produced many kinds of biosurfactants with different structure and physico-chemical properties. Based on structure diversity, biosurfactants can be classified into glycolipid, phospholipids, fatty acids, neutral lipids, lipopeptides, etc. (Bharali and Konwar, 2011). Biosurfactants may have several advantages over their chemically synthesized counterparts: high reliability and excellence even at extreme temperatures, pH, and salinities, lower toxicity, low critical micelle concentration (CMC) value, and biodegradablity. Those advantages make biosurfactants the most ideal substitute for the chemically synthesized surfactants (Plante et al., 2008).

Up to now, hundreds of biosurfactants have been identified, among which rhamnolipids (RLs) have been extensively studied. RLs are glycosides that are composed of a glycon and a aglycon part linked to each other via O-glycosidic linkage. The glycon part is composed of one or two rhamnose moieties linked to each other through α-1,2-glycosidic linkage (Edwards and Hayashi, 1965). Several kinds of different homologs of rhamnolipid have been reported (Abdel-Mawgoud et al., 2010), and mainly produced by bacteria of *Pseudomonas* and *Burkholderia* reported by far. Later, several other bacteria were also able to produce RLs as well, including *Renibacterium salmoninarum* (Christova et al., 2004), *Cellulomonas cellulans* (Arino et al., 1998), *Nocardioides* sp. (Vasileva-Tonkova and Gesheva, 2005), and *Tetragenococcus koreensis* (Lee et al., 2005).

RLs have a wide range of application including enhanced biodegradation of diesel and oil (Lang and Wullbrandt, 1999; Maier and Soberon-Chavez, 2000; Wang et al., 2008), and bioremediation of organic and heavy metal polluted sites (Mulligan, 2005). Besides, RLs are broadly used in the cosmetic industry for products such as moisturizers, toothpaste (Desai and Banat, 1997), and also be used in medical industry for their antimicrobial and antiviral properties (Ito et al., 1971; Lang and Wullbrandt, 1999; Haba et al., 2003). Those features make RLs a promising product.

Members of the genus *Dietzia* have been confirmed as alkane degraders (Rainey et al., 1995; Yumoto et al., 2002; von der Weid et al., 2007; Wang et al., 2011). In addition, *Dietzia* strains have the ability to degrade polycyclic aromatic compounds, including naphthalene (von der Weid et al., 2007), phenanthrene (Al-Awadhi et al., 2007), benzoate (Maeda et al., 1998), fluoranthene (Kumar et al., 2011). Recently, it was reported that two isolates of *Dietzia* can use alkane as the sole carbon to produce biosurfactants. Wax ester-like compounds were produced by *D. maris* WR-3 as biosurfactants (Nakano et al., 2011). Different kinds of biosurfactant were produced by *Dietzia* sp. DQ12-45-1b when using different alkanes as sole carbon source (Wang et al., 2013). However, the chemical characterization and properties of the biosurfactants have not been investigated in details.

In this paper, we reported an biosurfactant-producing strain *D. maris* As-13-3 isolated from deep sea hydrothermal field. When using *n*-hexadecane as the sole carbon source, it could produce di-rhamnolipid as biosurfactant. To further explore the mechanism of di-rhamnolipid biosynthesis, the genome of *D. maris* As-13-3 was sequenced and analyzed. The di-rhamnolipid biosynthesis-related genes were identified. These results bring new insights into the genetic and physiology of the genus *Dietzia*.

## Materials and methods

### Strain and cultivation

Strain As-13-3 was obtained from Marine Culture Collection of China (MCCC) with accession number as MCCC 1A00160, which was originally isolated from deep sea hydrothermal field environment of Southwest Indian Ocean (Chen and Shao, 2009). For the production of biosurfactant, it was cultivated in mineral salts medium (MSM; pH 7.4), which is composed of (NH_4_)_2_SO_4_, 10 g L^−1^; KCl, 1.1 g L^−1^; NaCl, 30 g L^−1^; FeSO_4_, 2.8 × 10^−4^ g L^−1^; KH_2_PO_4_, 3.4 g L^−1^; K_2_HPO_4_, 4.4 g L^−1^; MgSO_4_, 7.0 g L^−1^; yeast extract, 0.5 g L^−1^; trace elements solution, 0.5 ml L^−1^; and 2% (v/v) *n*-hexadecane as the sole carbon source. The trace elements solution contained (per liter): ZnSO_4_, 0.29 g; CaCl_2_, 0.24 g; CuSO_4_, 0.25 g; and MnSO_4_, 0.17 g, it was filtered with a 0.22-μm pore membrane. All chemicals were analytical grade unless specified, *n*-hexadecane was 98% pure and purchased from Fluka (Buchs, Switzerland). Bacteria were cultured in a 1L Chemostat (180 rev min^−1^) at 28°C for 7–10 days.

### Chemicals

All solvents and reagents used in this study are AR grade. The purity of all hydrocarbons used in this study is over 99% and checked by instrumental analysis.

### Nucleic acid extraction

Bacterial genomic DNA for strain As-13-3 was extracted using Axygen® AxyPrep™ Bacterial Genomic DNA Miniprep Kit (Axygen American).

A 2 mL sample was collected from each culture in the exponential phases using the RNA Bacteria Protect Reagent (Qiagen, Valencia, CA, USA). Total RNA was extracted using the RNeasy Mini Kit (Qiagen, Valencia, CA, USA) according to the manufacturer's protocol and subsequently treated with DNase I (Invitrogen, Carlsbad, CA, USA). The RNA yield was estimated using a Nanodrop UV Spectrometer (Thermo Scientific, Wilmington, DE, USA).

### Phylogenetic analysis

Strain As-13-3 was identified on the basis of its phylogenetic and physiological characteristics. The 16S rRNA gene was amplified from the genomic DNA. The 16S rRNA sequences were aligned and a phylogenetic tree was constructed using the Neighbor-Joining method.

### Surface activity test

Surface tension of the culture and its supernatant was measured using a DU Nouy ring tensiometer (model JZ-200A; Chengde Precision Testing Machine Co. Ltd., Hebei, China) according to McInerney et al. (1990). Pure water added with *n*-hexadecane at a final concentration of 2% (v/v) was used as a control. The surface tension value was the average of three repeats of the same culture.

### Effect of carbon sources on biosurfactant production

In order to investigate effect of carbon sources on the production of biosurfactant, strain As-13-3 was inoculated into MSM supplemented with different carbon source (2% v/v or m/v) including Glycerol, Glucose, Sodium citrate, Sodium acetate, Sodium pyruvate, *n*-Dodecane, *n*-Tetradecane, *n*-Hexadecane, Pristane, and Olive oil, and this culture was shaked (180 rev min^−1^) at 28°C for 7 days, then the OD_600_ and surface tension of culture broth were tested, respectively.

### Cell surface hydrophobicity

Cell surface hydrophobicity rates of strain As-13-3 were measured by bacterial adhesion to organic compounds according to Rosenberg (Rosenberg et al., 1980). Briefly, the cultures (5 ml) were centrifuged at 8000 rpm for 5 min. The cell pellets were washed twice with 3 ml AB buffer composed of K_2_HPO_4_·3H_2_O, 22.2 g; KH_2_PO_4_, 7.26 g; and MgSO_4_· 7H_2_O, 0.2 g, per liter H_2_O at pH 7.4, followed by resuspension in AB buffer to ensure the absorbance of the cell suspension at 600 nm of 0.5–0.6 (OD of the initial cell suspension). Hydrocarbons (0.2 ml), including *n*-Dodecane, *n*-Tetradecane, *n*-Hexadecane, Pristane, Toluene, and Paraffins, were added to 1.2 ml of the cell suspension, mixed thoroughly, transferred into a round-bottom test tube (i.e., 10 mm), and vortexed for 2 min. The mixture was then kept undisturbed at room temperature for 1 h, to achieve phase separation. The lower aqueous phase was then carefully removed and its OD values were measured at 600 nm (OD of the aqueous phase). Hydrophobicity was expressed as the percentage of adherence to the hydrocarbon, which was calculated as follows: 100 × (1-OD of the aqueous phase/OD of the initial cell suspension). The cells grown in LB medium were used as a negative control.

### Emulsification index (E_24_)

The emulsification index (E_24_) of supernatant samples was determined according to Burgos-Díaz et al. (2011). Generally speaking, 2 mL of organic matter was added to 2 mL of culture supernatant, then vortexed at a high speed for 2 min. The mixture was then allowed to stand still for 24 h prior to measurement. Emulsification activity was defined as the height of the emulsion layer divided by the total height and expressed as percentage. Data represent the mean of three independent experiments.

### Biosurfactant extraction and purification

The biosurfactant extraction and purification process was done according to Qiao and Shao (2010). The culture broth was first centrifuged for 20 min at 12,000 rpm at 4°C, a hydrophobic layer floating on the surface was scraped out and washed with three volumes of hexane to remove alkanes. Then the crude material was extracted with chloroform/methanol (v/v 1:1) for three times. The solvent was then removed by rotary evaporation at 35°C under reduced pressure, and the crude extract was stored at −20°C until subjected to further purification.

The crude extract was further purified using a silica gel column, the column was washed with the following solvent systems with increasing polarity: chloroform; methanol/chloroform (95:5, v/v); methanol/chloroform (90:10, v/v); methanol/chloroform (80:20, v/v); 100% methanol. The eluates demonstrated to have the highest surface activity was further separated by a Sephadex LH-20 gel column. The velocity of flow was about 12 s per drop, and flow solvent was chloroform: methanol (1:1, v/v). After purification by using Sephadex LH-20 gel column, the eluates exhibited a high surface activity were collected and pooled together for further purification.

### Thin layer chromatography (TLC) analysis

The eluate was further analyzed by TLC on silica gel F_254_ with the following solvent system: chloroform/methanol/water (80:15:2, v/v/v).

Four reagents were used to test the category of the surfactant: phenol/sulfuric acid reagent for glycolipids; 0.2% ninhydrin reagent for lipopeptides; cobalt chloride/acetone reagent for phospholipids; and bromocresol green for lipid-organic acids.

### High performance liquid chromatography (HPLC) analysis

HPLC analysis of the biosurfactant was done according to Bharali and Konwar with slight modification (Bharali and Konwar, 2011). The components of partially purified biosurfactant were fractionated using a gradient elution HPLC (Waters 2487) with a UV and Evaporative Light-scattering Detector (ELSD). A Chromolith Fast Gradien RP-18e, with a dimension of 50–3 mm was used. An acetonitrile-water gradient containing 0.1% trifluoroacetic acid was used, starting with 5% B increase to 100% B within 0.8 min, 100% B for 1.1 min. The mobile phase was kept at a flow rate of 1.5 ml/min and the sample injection volume was 10 μl. All fractions eluted from the HPLC column were collected at different retention times. The fractions were then evaporated at room temperature to remove all of the solvent part to obtain a purified biosurfactant. The fraction having the height reduction in surface tension of water was selected and further characterized.

### Chemical characterization of the purified biosurfactant: LCQ-MS and NMR

Mass spectrometer (MS) characterization and detection of the biosurfactant was carried out according to Qiao and Shao (2010). The purified biosurfactant was characterized by using a LCQ quadrupole ion-trap MS (Finnigan MAT, San Jose, CA, USA) with electrospray ionization (ESI). Standard solutions and samples were infused into the mass spectrometer at a flow rate of 10 μL min^−1^. In the ESI source, the nitrogen sheath and auxiliary gas flows were maintained at 50 and 5, respectively; these refer to arbitrary values set by the software. The heated capillary temperature was 275°C, and the spray voltage was set to 5000 V. The positive ion mode was used and scans were run over the 100–1000 m/z range.

The purified biosurfactant was dissolved in denatured chloroform (DCCl_3_) and analyzed with nuclear magnetic resonance (NMR). The NMR spectra were recorded on Bruker 400 MHz NMR spectrometer at 25°C.

### Determination of critical micelle concentration (CMC)

For the determination of CMC value, different aqueous concentrations of biosurfactants were prepared. Surface tensions were measured using a DU Nouy ring tensiometer as described before. And then the surface tension-concentration plots were drew, the CMC value was determined as the intersection of linear component of the curve drawn between the surface tension and the concentration of biosurfactant, and it was expressed in 5 mg l^−1^. For calibrating the instrument, it was subjected to the determination of surface tension of the pure water at 25 ± 1°C (Makkar and Cameotra, 1997).

### Degradation of hydrocarbon and GC-MS analysis

The strain As-13-3 was inoculated into mineral salts medium MSM supplemented with different carbon source (1% v/v), and this culture was shaking (180 rev min^−1^) at 28°C for 10 days to test the hydrocarbon degradation rate. Non-inoculated flasks were served as controls. Residual hydrocarbons were extracted three times from the cultures by shaking vigorously with an equal volume of *n*-hexane. The *n*-hexane was concentrated by rotary evaporation at 35°C under reduced pressure, then the concentrated extracts were subjected to gas chromatography-mass spectrometry (GC-MS). In GC-MS analyses, the GC temperature program was 70–300°C at 4°C min^−1^ with a 15-min hold time. In GC-FID analysis, the program was 50–100°C at 6°C min^−1^, then to 300°C at 4°C min^−1^ with a 15-min hold time. Helium was used as the carrier gas at a constant pressure of 180 kPa. Also standard curve of different hydrocarbon was drawn under the same condition, and the concentration of residue hydrocarbon was calculated according to the standard curve.

The percentage of hydrocarbon degradation was calculated as follows: 100 × (the hydrocarbon concentration of experimental group/the hydrocarbon concentration of the control group). The experimental data are presented in terms of arithmetic averages of at least three replicates, and the standard deviations are indicated by error bars.

### Sequencing of the genome of strain As-13-3

The genome sequencing of strain As-13-3 was performed with a Solexa paired-end sequencing technologies (Bentley et al., 2008). Genomic libraries containing 3-kb inserts were constructed. A total of 5,545,034 paired-end reads were generated with an Illumina Solexa Genome Analyzer IIx (Illumina, San Diego, CA) to reach a depth of 182.9-fold coverage, and 69.9% of the reads were assembled into 123 scaffolds by using the Burrows–Wheeler Alignment (BWA) tool (Li and Durbin, 2010), including 188 non-redundant contigs. Protein encoding genes were predicted by Glimmer 3.0 (Delcher et al., 2007). The analysis of the genome was performed as described previously (Feng et al., 2008; Li et al., 2011). The genome sequence was also submitted to the Integrated Microbial Genomes (IMG) server (http://img.jgi.doe.gov) of the Joint Genome Institute (JGI) for deep analysis and genome comparison (Markowitz et al., 2010).

### Real-time PCR

Approximately 4 μg of RNA was reversely transcribed using 20 ng of random primers (Invitrogen, Carlsbad, CA, USA) and PrimeScript™ Reverse Transcriptase (Takara, Dalian, China). Control reactions were performed without reverse transcriptase to verify the absence of genomic DNA.

The primers for real-time PCR were designed using Primer Premier 5.0 (http://www.premierbiosoft.com/). The primer sequences are listed in Table S1 and were synthesized by Invitrogen (Shanghai, China). Quantitative real-time PCR was performed using IQ™ SYBR Green Supermix and the IQ™ 5 Multicolor Real-time PCR Detection System (Bio-Rad, California, USA). The reactions were performed in 96-well optical plates sealed with optical caps. The total reaction volume of 25 μl contained 12.5 μl of 2X SYBR® Green PCR Supermix (Bio-Rad, California, USA), the DNA template, primers at an optimized concentration and sterile water. The following program was utilized: 2 min at 50°C (uracil-N-glycosylase activation), 10 min at 95°C (activation of Taq polymerase) and 40 cycles of denaturation (10 s at 95°C), annealing and elongation (30 s at 56–61°C). Fluorescence data were acquired at the end of the elongation step. The specificity of the accumulated products was verified through melting curve analysis. In all of the experiments, appropriate negative controls were subjected to the same procedure to detect any possible contamination. The size and purity of the obtained amplicon and the absence of dimer formation were assayed through conventional agarose gel electrophoresis.

### Statistical analysis

The 16S rRNA housekeeping gene was used as a reference gene to normalize gene expression in strain As-13-3. The relative fold change in mRNA quantity was calculated for the gene of interest in each sample using the ΔΔCt method. For each RNA preparation, at least three independent real-time PCR experiments were conducted. Data were analyzed by unpaired two-tailed Student's *t*-tests or One-Way ANOVA, followed by Tukey's multiple comparison test with GraphPad Prism software (San Diego, CA, USA). Data were expressed as mean ± SD derived from at least three independent experiments. Differences were considered significant at *P* < 0.05.

### Sequence accession numbers

The sequences of the di-rhamnolipid biosynthesis-related genes of *D. maris* As-13-3 have been deposited in the NCBI database with the following accession numbers: KP202067 through KP202092.

## Results and discussion

### The characterization of the strain As-13-3

**Figure 1:**
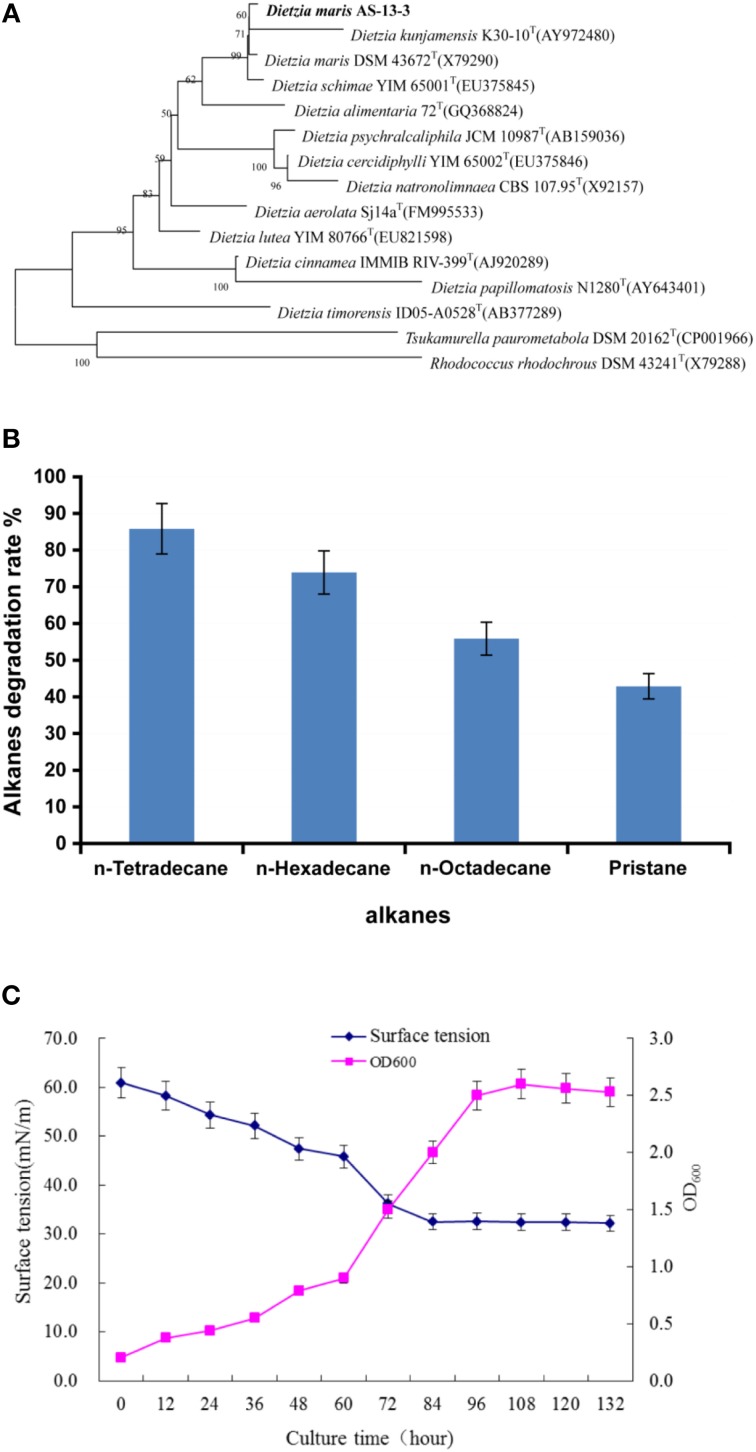
**The characterization of the strain As13-3**. **(A)** Neighbor-Joining tree showing the phylogenetic positions of strain AS-13-3 and representatives of some other related taxa, based on 16S rRNA gene sequences. Bootstrap values (expressed as percentages of 1000 replications) are shown at branch points. Bar, 0.005 nucleotide substitution rate (Kunc) units. **(B)** Degradation of individual *n*-alkanes by AS-13-3. **(C)** Relationship between cell growth and biosurfactants production during the growth of strain AS-13-3. Data are the means of three separate experiments. The error bars represent the S.D.

Phylogenetic analyses showed that strain As-13-3 formed a stable clade with the type strains of all species in the genus *Dietzia*, and the highest 16S rRNA sequence similarity of 99.22% with *Dietzia maris* DSM 43672T(X79290) (Figure 1A). Strain As-13-3 can utilize short-chain and middle-chain *n*-alkanes (from C_8_ to C_20_, data not shown), and vigorous degradation occurred from C_14_ to C_18_ and pristane (Figure 1B). Notably, strain As-13-3 can also utilize branched-alkanes pristane (2,6,10,14-tetramethyl-pentadecan), and the degradation rate was 42.87% in 10 days (Figure 1B). The cell surface hydrophobicity (CSH) of strain As-13-3 against different kinds of hydrocarbon substrate was also tested. The results showed that strain As-13-3 have the highest CSH toward toluene with hydrophobicity of 73.3%, and a relative high CSH toward *n*-hexadecane and *n*-tetradecane with the CSH of about 60%, respectively (Table 1). In contrast, strain As-13-3 exhibits a weak hydrophobicity toward dodecane and pristane with hydrophobicity of 40.7 and 38.8%, respectively (Table 1).

**Table 1:** **The cell surface hydrophobicity of *D. maris* AS-13-3 grown on various hydrocarbon substrate^a^**.

**Hydrocarbon**	***n*-Dodecane**	***n*-Tetradecane**	***n*-Hexadecane**	**Pristane**	**Toluene**	**Paraffins**
Hydrophobicity	40.7%	63.9%	61.2%	38.8%	73.3%	51.8%

a *Data are the mean of three separate experiments*.

Alkanes and non-alkane carbon sources were used to monitor the biosurfactant production. The results demonstrated that strain As-13-3 produced biosurfactant only in the presence of alkanes such as *n*-tetradecane, *n*-hexadecane, and Pristane (Table 2). Otherwise, the surface tension of culture broth had no notable variation when using simple carbon sources such as glucose and glycerol (Table 2). Using *n*-hexadecane as the sole carbon source, the culture broth surface tension decreased during the cultivation period, reaching 33.1 mN m^−1^ after 5 days of cultivation, and then maintained at a constant level (Figure 1C).

**Table 2:** **Effect of carbon sources on bacterial growth and biosurfactant production^a^**.

**Carbon sources**	**OD_600_ of culture broths**	**Surface tension of culture broth (mN/m)**
Glycerol	2.10	56.4
Glucose	1.10	55.4
Sodium citrate	1.27	61.1
Sodium acetate	2.10	59.1
Sodium pyruvate	1.35	59.1
*n*-Dodecane	2.04	40.1
*n*-Tetradecane	1.66	35.6
*n*-Hexadecane	1.22	33.2
Pristane	0.805	34.0
Olive oil	4.01	41.4
Control	0	62.0

a *Data are the mean of three separate experiments*.

### Biosurfactant structure and characteristics

**Figure 2:**
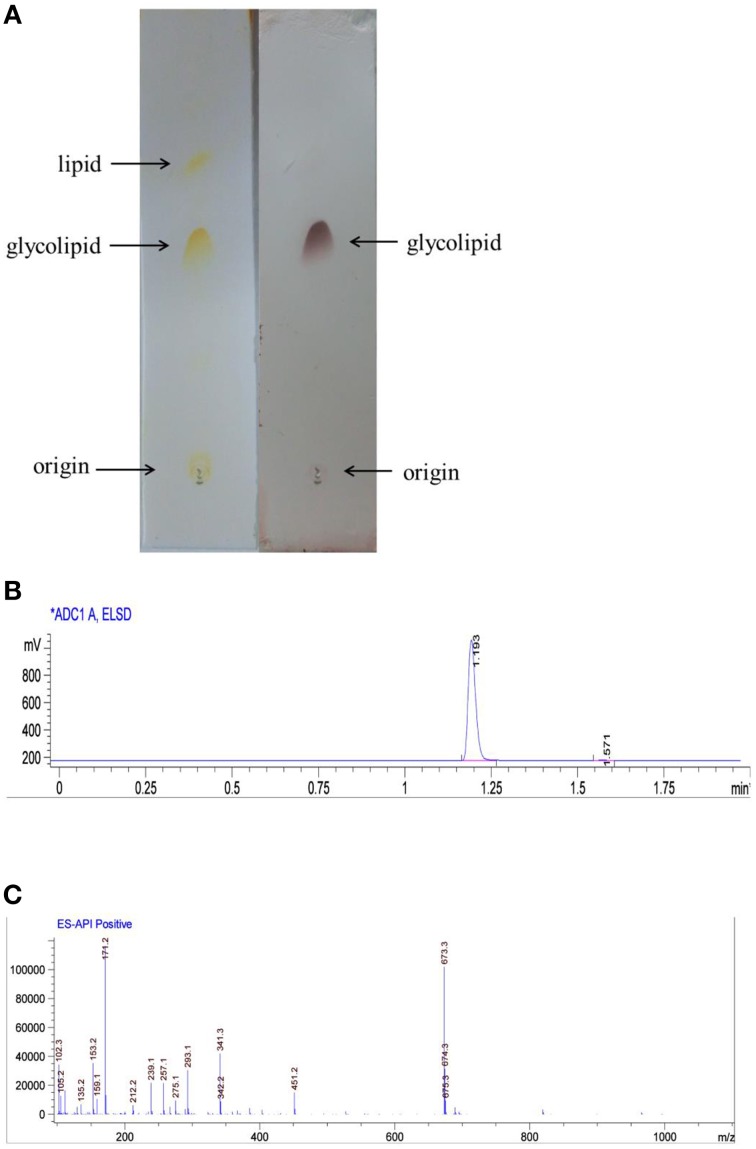
**Biosurfactant purification and structural characterization. (A)** Thin layer chromatogrpahy analysis of glycolipid produced by AS-13-3 developed with chloroform–methanol–water(85:15:2,v/v/v). detected with iodine (plate A) and phenol–sulfuric acids (plate B). **(B)** HPLC analysis of biosurfacant produced by strain AS-13-3(ELSD detector). **(C)** Molecular mass spectra of the glycolipid produced by strain AS-13-3.

The partially purified biosurfactant that demonstrated to have the highest surface activity was further analyzed by thin layer chromatography and visualized with specific reagents. After phenol/sulfuric acid reagent staining a black–brown prominent spot was observed, the biosurfactant was identified as glycolipid (Figure 2A). The glycolipid product was future subjected to HPLC analysis and preparation, the retention time of the purified biosurfactant was 1.193 min (Figure 2B). LCQ-MS results revealed molecular ion peak with molecular masses of 673.3, and the glycolipid was assigned to the protonated molecular ion and to the adduct of sodium ions [M+Na^+^] (Figure 2C). Thus, the molecular weight of the glycolipid is 650.

**Figure 3:**
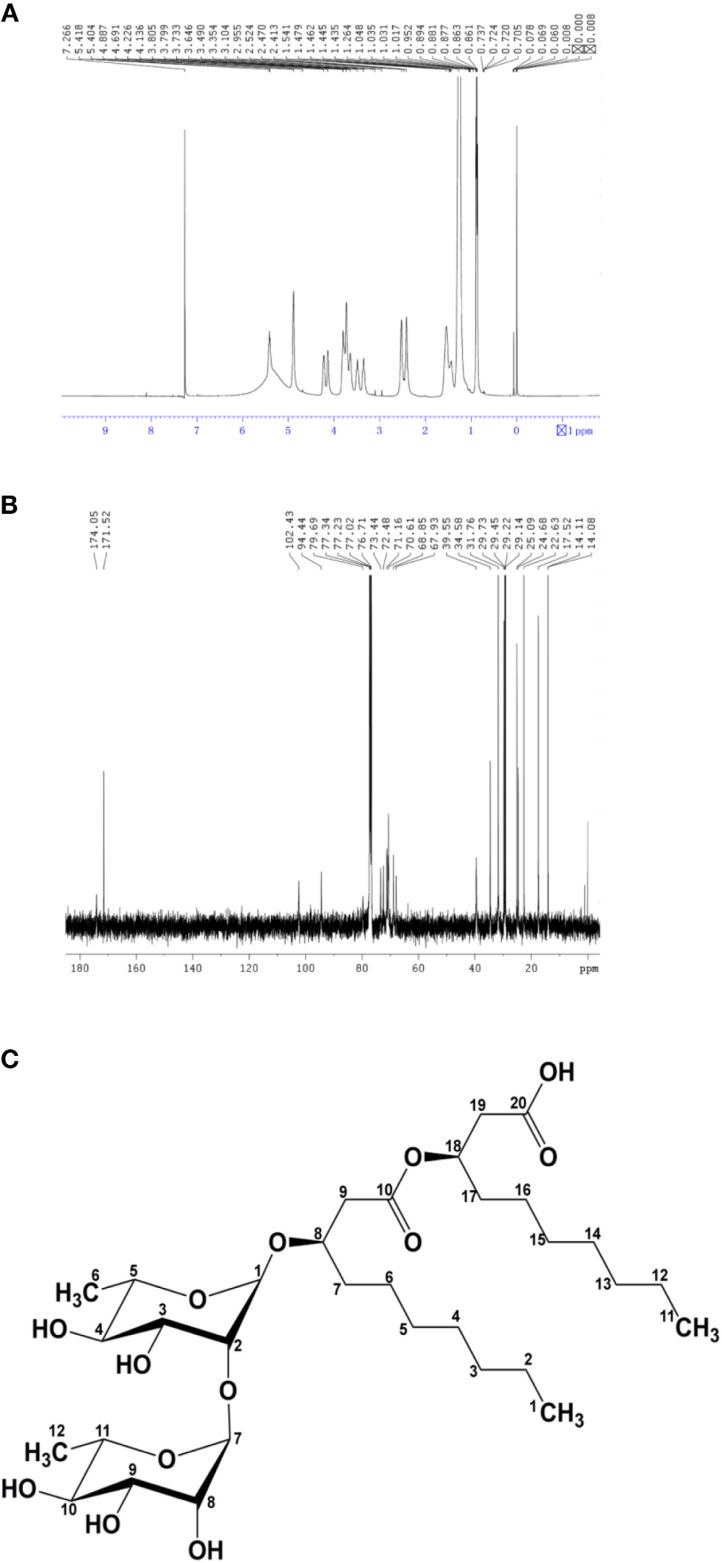
**Biosurfactant structural ananlyisis. (A)** ^1^H nuclear magnetic resonance spectrum of the lipopetide produced by strain AS-13-3. **(B)** ^13^C nuclear magnetic resonance spectrum of the lipopetide produced by strain AS-13-3. The chemical groups represented by each peak are given in the **Table 4**. **(C)** Molecular structure of the di-rhamnolipid produced by strain AS-13-3.

The molecular structure of the glycolipid was further analyzed by ^1^H-NMR (Figure 3A), ^13^C-NMR (Figure 3B) and other 2D-NMR spectroscopies. The chemical shifts of the compound are summarized in **Table 4**. In ^1^H-NMR, characteristic chemical shifts 1.21 ppm showed the presence of –CH_3_ in sugar portion, and similarly 4.89 ppm for 1′-H, 3.63–4.23 ppm for 2′,3′,4′,5′-H. In fatty acid portion, 0.89 ppm in ^1^H-NMR was supposed to be–CH_3_ and peaks at 0.88–1.28 ppm revealed alkyl group in fatty acid portion. Besides, the chemical shifts at 2.48, 4.14, and 5.35 ppm represents hydrogen atom for–CH_2_–COO–, –O–C–H, and –COO–CH_2_, respectively. In the ^13^C-NMR spectrum, the peak at 174.1 and 171.5 ppm were assigned to be carbonyl groups. All parameters such as ^1^H-NMR and ^13^C-NMR chemical shifts and other 2D-NMR spectroscopies indicated the molecular structure as that of L-rhamnosyl-L-rhamnosy-β-hydroxydecanoate (di-rhamnolipid) (Figure 3C).

The di-rhamnolipid showed satisfying emulsification ability with all the substrate tested including *n*-Hexane, *n*-Hexadecane, and Pristane, and the E_24_ value is about 50% (Table 3). The maximum emulsification index was obtained with cyclohexane and diesel, and the E_24_ value is about 60% (Table 3). The CMC value of the di-rhamnolipid is 120 mgL^−1^ (Figure S1). The surface tension of water was reduced by di-rhamnolipid from 74 ± 0.2 to 38 ± 0.2 mN m^−1^ (Figure S1). The CMC value and surface tension of di-rhamnolipid produced by strain As-13-3 is a little higher than *Pseudomonas aeruginosa* species produced RLs. In 2011, Bharali reported a *P. aeruginosa* strain OBP1 which could produce di-rhamnolipid, it reduced the surface tension of water to 31.1 mN m^−1^ with a CMC value of 45 mg L^−1^ (Bharali and Konwar, 2011). Also, it was reported that the RLs produced by *P. aeruginosa* species could reduce the surface tension of water from 72 to 29 mN m^−1^ with a CMC value in the range of 5–60 mg L^−1^ (Van Dyke et al., 1993). However, the di-rhamnolipid produced by strain As-13-3 exhibited better performance of surface actives compared with rhamnolipids produced by *Burkholderia thailandensis* which reduce the surface tension of water to 42 mN m^−1^ and displaying the CMC value of 225 mg L^−1^ (Dubeau et al., 2009).

**Table 3:** **The emulsification index (E_24_) of the produced biosurfactant by AS-13-3 grown on various hydrocarbon substrate^a^**.

**Hydrocarbon**	**Toluene**	***n*-Hexane**	**Cyclohexane**	***n*-Hexadecane**	**Pristane**	**Diesel**
**E_24_**	60.29%	55.88%	63.64%	55.88%	54.29%	63.64%

a *Data are the mean of three separate experiments*.

**Table 4:** **^1^H-NMR and ^13^C-NMR spectrum of purified biosurfactant by strain AS-13-3**.

**Hydrogen atoms**	**^1^H-NMR (ppm)**	**Carbon atoms (Sugar-portion)**	**^13^C-NMR (ppm)**
**SUGAR-PORTION**			
1′-H	4.89	1′-C,7′-C	102.21, 94.46
CH_3_	1.21	2′-C,8′-C	73.46
2′,3′,4′,5′-H	3.63-4.23	3′-C,9′-C	–
2′,3′,4′,5′-H		4′-C,10′-C	–
		5′-C,11′-C	67.92
		6′-C,12′-C	17.55
**FATTY ACID-PORTION**			
CH_3_	0.89	1-C,11-C	14.11
CH_2_	1.20–1.45	2-C,12-C	34.81
CH_2_CO_2_	2.48	3-C,13-C	–
CO_2_CH	5.35–5.42	4-C,14-C	–
O–CH	4.14	5-C,15-C	–
		6-C,16-C	–
		7-C,17-C	22.66
		8-C,18-C	73.46–67.92
		9-C,19-C	39.52
		10-C,20-C	174.14, 171.53

### Biosynthesis pathway of rhamnolipid in strain As-13-3

Rhamnolipids (RLs) is the most intensively studied biosurfactant, and the biosynthesis have been clarified by many reports (Burger et al., 1963; Rehm et al., 2001; Deziel et al., 2003; Zhu and Rock, 2008). It was reported that the substrates for the biosynthesis of rhamnolipid were glucose for the biosynthesis of rhamnose moiety and acetyl-CoA for the biosynthesis of lipid moiety (Koga, 1997; Madduri et al., 2001; Abdel-Mawgoud et al., 2011). The genes that played important roles in RLs biosynthesis process were also reported (Ochsner et al., 1994; Urs et al., 1994; Campos-Garcia et al., 1998; Messner, 1999; Soberón-Chávez, 2004). Generally speaking, RLs biosynthesis can be divided into three parts: biosynthesis of the fatty acid; sugar moieties and link the sugar and lipid (Gunther et al., 2005; Abdel-Mawgoud et al., 2011).

**Figure 4:**
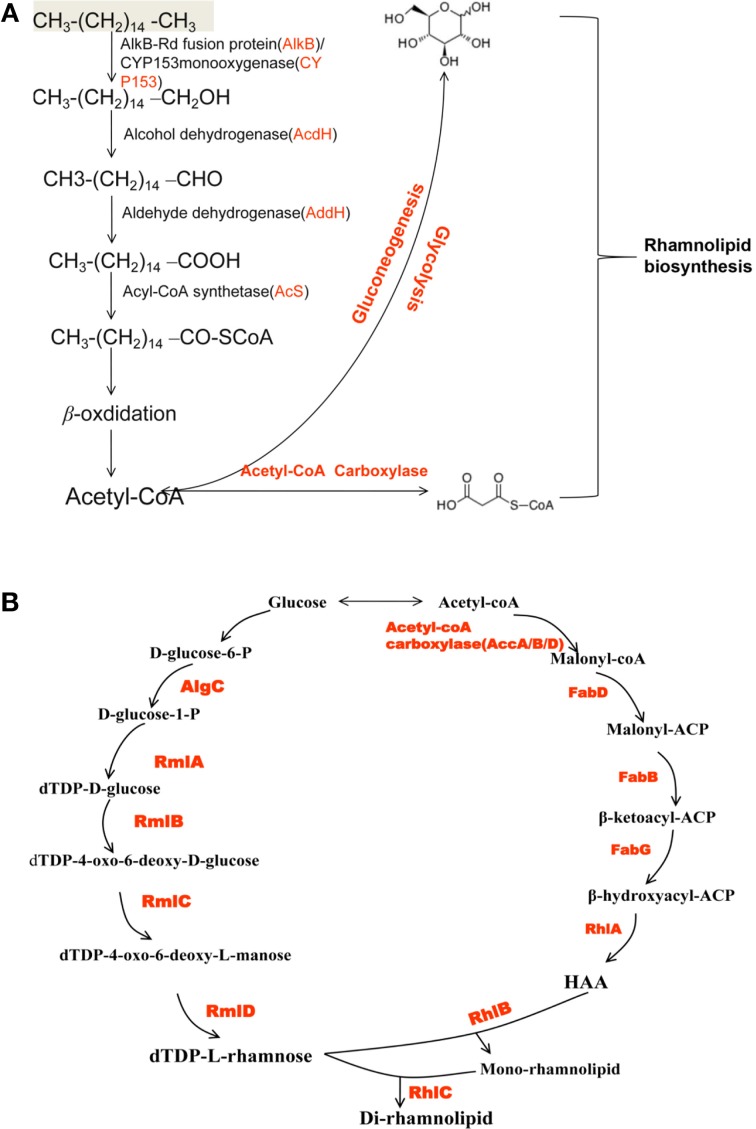
**Schematic overview of rhamnolipid biosynthesis pathways in strain AS-13-3. (A)** The alkanes degradation pathways. AlkB: AlkB-Rub fusion alkane monooxygenase; CYP153: CYP153 alkane monooxygenase gene; acdH: alcohol dehydrogenase gene; addH: aldehyde dehydrogenase; acS: acyl-CoA synthetase. **(B)** The di-rhamnolipid biosynthesis pathways. AlgC: phosphomannomutase; RmlA: glucose-1-phosphate thymidylyltransferase; RmlB: dTDP-D-glucose 4,6-dehydratase; RmlC: dTDP-4-dehydrorhamnose 3,5-epimerase; RmlD: dTDP-4-dehydrorhamnose reductase; FabD: malonyl-CoA:ACP transacylase; FabH, FabB and FabF: b-ketoacyl-ACP synthetases; FabG: NADPH-dependent b-ketoacyl-ACP reductase; HAA: 3-(3-hydroxyalkanoyloxy) alkanoic acid; RhlA: 3-(3-hydroxyalkanoyloxy) alkanoate synthetase; RhlB: rhamnosyltransferase; RhlC: rhamnosyltransferase.

**Figure 5:**
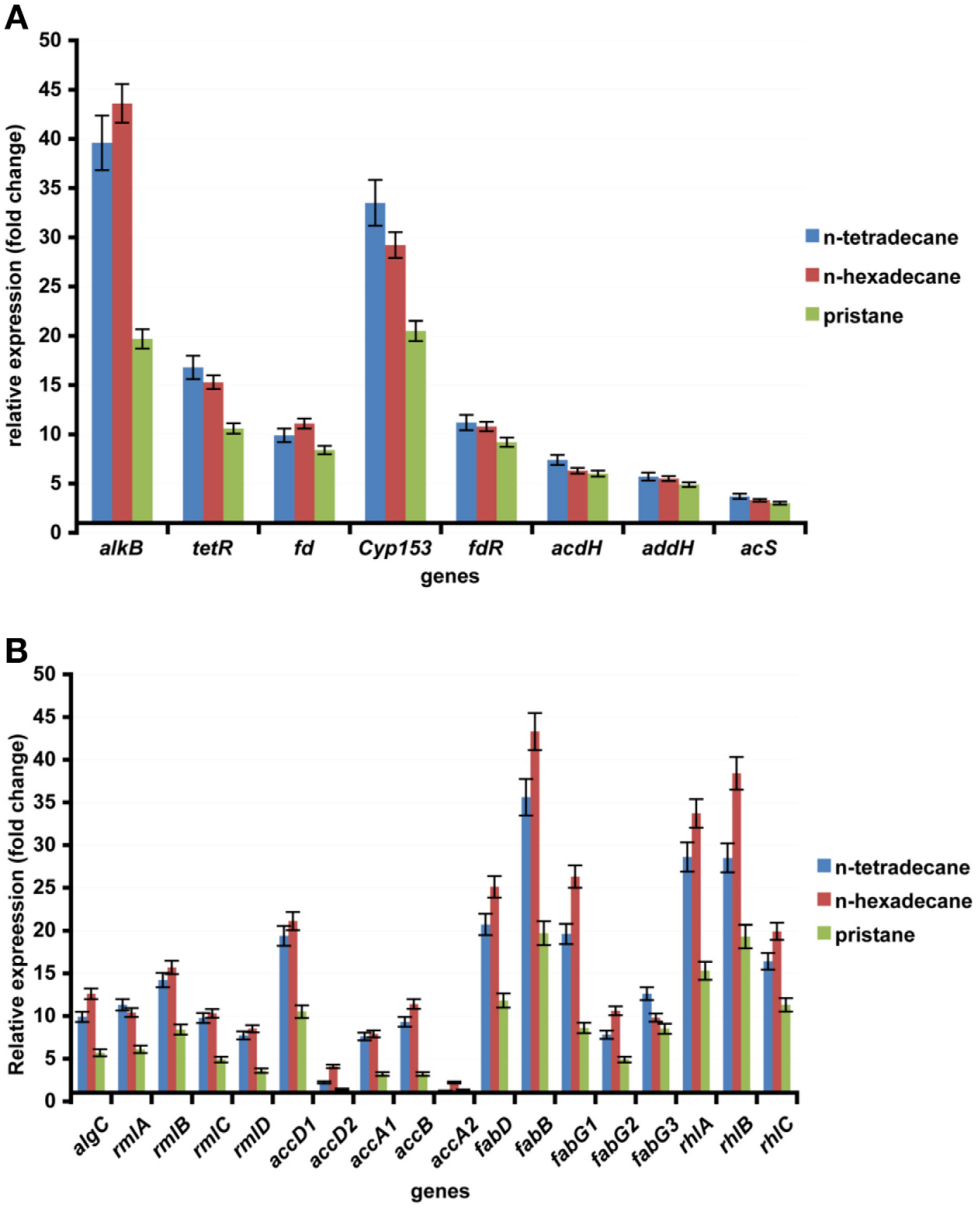
**The expression of alkanes degradation and biosynthesis of rhamnolipid related gene in the AS-13-3 strains grown on various carbon sources and in control cells grown on sodium acetate**. The data are presented as the mean of three independent experiments. The error bars represent the S.D. **(A)** The expression of *alkB*, *cyp153, tetR, fd, fdR, acdH, addH*, and *acS* in AS-13-3 cells. **(B)** The expression of *algC, rmlA, rmlB, rmlC, rmlD, accA1, accA2, accB, accD1, accD2, fadD, fadB, fagG1, fadG2, fadG3, rhlA, rhlB*, and *rhlC* in AS-13-3 cells.

**Figure 6:**
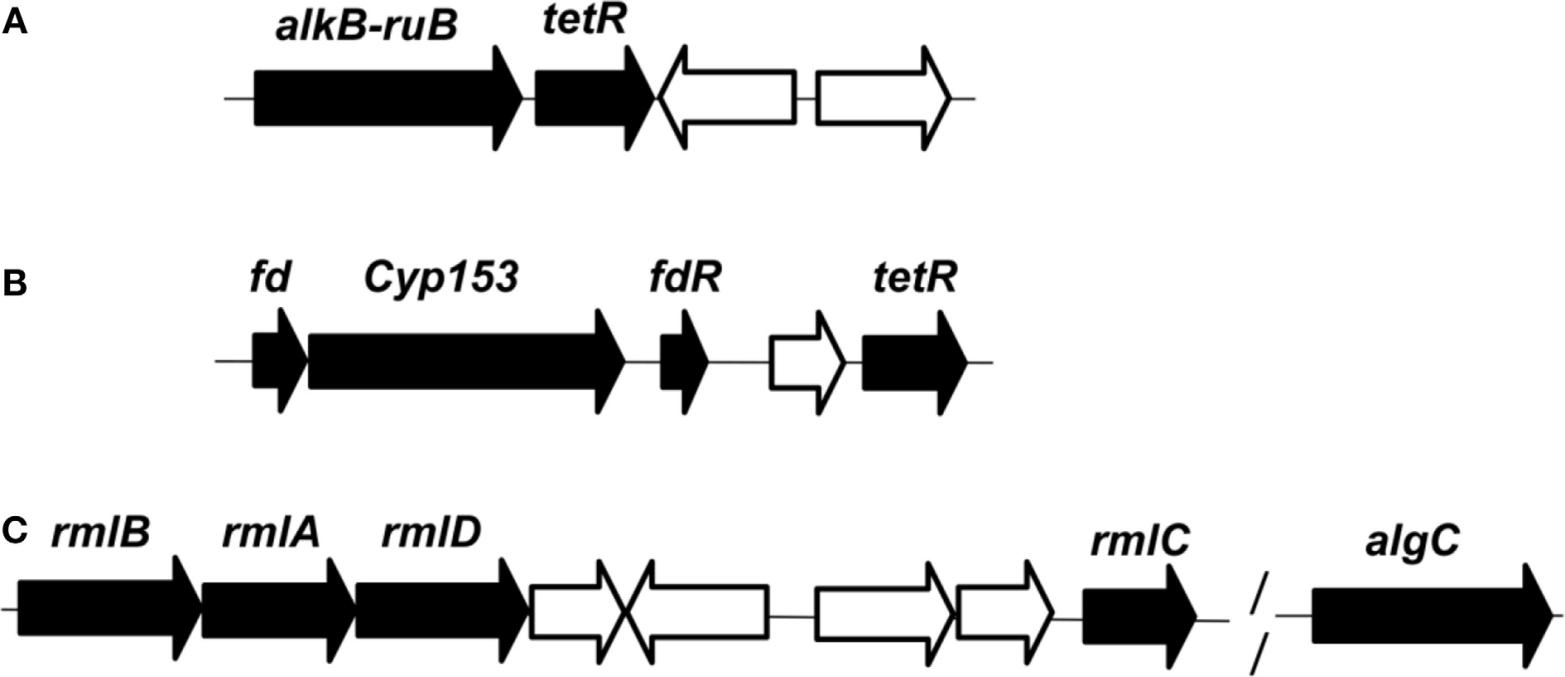
**Organization of the genes involved in the degradation of *n*-alkanes and synthesis of dTDP-L-rhamnose in genome of *D.maris* AS-13-3**. **(A):** *alkB-rub* fusion gene. **(B)**: cyp-153 gene. **(C)**: rmlA/B/C/D and algC gene. Black, proteins involved in the degradation pathway or the synthesis pathway. White, proteins are not involved in the degradation pathway or the synthesis pathway. *alkB-rub*: FLAG-tagged AlkB-Rd fusion protein; *tetR*: TetR family transcriptional regulator; *fd*: 2Fe-2S ferredoxin protein; *cyp153*: cytochrome P450 153A16 monooxygenase; *fdR*: ferredoxin reductase;*rmlA*:glucose-1-phosphate thymidylyltransferase; *rmlB*:dTDP-glucose 4,6-dehydratase; *rmlC*:dTDP-4-dehydrorhamnose3,5-epimerase; *rmlD*:dTDP-4-dehydrorhamnosenose reductase; *algC*: phosphoglucomutase.

In this report, *n*-hexadecane was served as the sole carbon source in the medium, indicating that the raw material for the biosynthesis of rhamnolipid is derived from *n*-hexadecane. The genome of strain As-13-3 revealed a complete pathway for the biodegradation of alkanes by terminal oxidation (Figure 4A). The pathway genes including alkane hydroxylase genes: AlkB-Rub fusion protein gene (*alkB*) and CYP153 alkane monooxygenase gene (*cyp153*), alcohol dehydrogenase gene (*acdH)*, aldehyde dehydrogenase gene (*addH*), acyl-CoA synthetase gene (*acS*), and TCA cycle and gluconeogenesis related genes (data not shown) (Table 5). Quantitative real-time PCR (Q-PCR) analysis showed that *alkB* and *cyp153* expression were strongly induced by *n*-Tetradecane, *n*-Hexadecane, and pristane, respectively (Figure 5A). The *n*-Hexadecane increased *alkB* and *cyp153* by 49.6- and 29.2-fold, respectively. The *tetR*, fd, *fdR*, *acdH*, *addH*, and *acS* gene were induced moderately by *n*-Tetradecane, *n*-Hexadecane, and pristane, respectively (Figure 5A). Interestingly, the arrangement of alkane hydroxylase genes on the chromosome of strain As-13-3, with the presence of *alkB-rub* gene or *cyp153* gene, are very similar to that previously reported in the related *Dietzia* sp.DQ12-45-1b (Figure 6) (Nie et al., 2011, 2014).

**Table 5:** **The alkanes degradation and rhamnolipids biosynthesis related genes identified in genome of *D. maris* AS-13-3**.

**Gene name**	**Functional description**	**Organism**	**Identity**	**GenBank ID**
*alkB*	AlkB-Rd fusion protein	*Dietzia cinnamea* P4	100%	KP202090
*tetR*	TetR family transcriptional regulator	*Dietzia* sp. E1	95%	KP202073
*fd*	Ferredoxin reductase	*Dietzia cinnamea* P4	100%	KP202087
*cyp153*	Cytochrome P450 153A16	*Dietzia cinnamea* P4	99%	KP202088
*fdR*	2Fe-2S ferredoxin	*Dietzia cinnamea* P4	100%	KP202089
*acdH*	Alcohol dehydrogenase	*Dietzia cinnamea* P4	96%	KP202082
*addH*	Aldehyde dehydrogenase	*Dietzia cinnamea* P4	94%	KP202081
*acS*	Acyl-CoA synthetase	*Dietzia cinnamea* P4	91%	KP202084
*algC*	Phosphomannomutase	*Dietzia cinnamea* P4	85.65%	KP202070
*rmlC*	dTDP-4-dehydrorhamnose 3,5-epimerase	*Dietzia cinnamea* P4	97%	KP202076
*rmlA*	Glucose-1-phosphate thymidylyltransferase	*Dietzia cinnamea* P4	62%	KP202079
*rmlB*	dTDP-glucose 4,6-dehydratase	*Dietzia cinnamea* P4	95%	KP202080
*rmlD*	dTDP-4-dehydrorhamnose reductase	*Rhodococcus equi* ATCC 33707	92%	KP202078
*accD1*	Acetyl-CoA carboxylase	*Dietzia cinnamea* P4	97%	KP202072
*accD2*	Acetyl-CoA carboxylase	*Rhodococcus opacus* PD630	77%	KP202086
*accA1*	Acyl-CoA carboxylase alpha chain	*Dietzia cinnamea* P4	94%	KP202085
*accB*	Acyl-CoA carboxylase beta chain	*Dietzia cinnamea* P4	97%	KP202074
*accA2*	Acyl-CoA carboxylase alpha chain	*Dietzia cinnamea* P4	98%	KP202075
*fabD*	ACP-S-malonyltransferase	*Dietzia cinnamea* P4	95%	KP202083
*fabB*	Beta-ketoacyl-ACP synthase I	*Dietzia cinnamea* P4	89%	KP202067
*fabG1*	3-Ketoacyl-ACP reductase	*Dietzia cinnamea* P4	98%	KP202068
*fabG2*	3-Ketoacyl-ACP reductase	*Dietzia cinnamea* P4	98%	KP202069
*fabG3*	3-Ketoacyl-ACP reductase	*Dietzia cinnamea* P4	97%	KP202077
*rhlA*	3-(3-Hydroxyalkanoyloxy)alkanoic acids (HAAs) synthase	*Burkholderia cenocepacia*	96%	KP202092
*rhlB*	Alpha-3-L-rhamnosyl transferase	*Dietzia cinnamea* P4	92%	KP202071
*rhlC*	Alpha-3-L-rhamnosyl transferase	*Dietzia alimentaria*	90%	KP202091

The lipid moiety of RL was previously reported to be generated through the classical pathway of fatty acid synthesis (Zhu and Rock, 2008). In genome of strain As-13-3, we also found fatty acid synthetases of type-II (FAS II) pathway, the key genes include *accA*, *accB*, *accD*, *fabB, fabD, and fabG* (Table 5). The acetyl-CoA for substrate via a series of enzymatic catalytic reactions (the FAS II pathway) changes into the β-hydroxy fatty acids (HAA), and the HAA was future used in the biosynthesis of RLs (Figure 4B). The expression profiles of three *fabG*, *fabB*, and *fabD* were also induced significantly by *n*-Tetradecane, *n*-Hexadecane, and pristane (Figure 5B); as well as the genes involved in fatty acid synthetases, like *accA1*, *accB*, and *accD1* (Figure 5B); however, *accA2* and *accD2* were not sensitive to the presence of alkanes (Figure 5B).

Almost all the genes involved in the biosynthesis of dTDP-L-rhamnose were also found based on genome analysis. These genes including *algC*, *rmlA*, *rmlB*, *rmlC*, and *rmlD*. Q-PCR analysis showed that these genes were induced significantly by *n*-Tetradecane, *n*-Hexadecane, and pristane, respectively (Figure 5B). Olvera et al. reported that *algC* mutant seriously impacted the production of RLs in *P. aeruginosa* (Olvera et al., 1999). Thus, in the production of rhamnolipid, *algC* plays an important role.

Glycosyltransferase constitutes a large family of enzymes that are involved in the biosynthesis of oligosaccharides, polysaccharides, glycoproteins, glycolipids and other glycoconjugates (Paulson and Colley, 1989; Breton et al., 2006). They catalyze the sugar residues from an activated donor substrate into saccharide and non-saccharide acceptors (Breton et al., 2006). In the biosynthesis of rhamnolipids, rhamnosyltransferase participates in the final steps, which link the dTDP-L-rhamnose and lipidic moiety of RLs to yield the final products (RLs), those genes including *rhlA*, *rhlB*, and *rhlC* (Rahim et al., 2001; Dubeau et al., 2009; Abdel-Mawgoud et al., 2011). In this report, *rhlA*, *rhlB*, and *rhlC* encoding rhamnosyl transferases that linked dTDP-L-rhamnose and β-hydroxy fatty acids (HAA) together were identified, respectively (Figure 4B). Q-PCR analysis showed that the *rhlA*, *rhlB*, and *rhlC* genes were induced significantly by all the tested alkanes, respectively (Figure 5B).

According to above experiment results, strain As-13-3 can use different hydrocarbons including branched alkane pristane as the raw material to produce biosurfactant (Figure 4). Q-PCR analysis showed that almost all above-mentioned genes were induced significantly by alkanes (Figure 5). In the case of strain *Dietzia sp*. DQ12-45-1b, it can produce kinds of biosurfactants corresponding to the presence of *n*-alkanes (Wang et al., 2013). When using *n*-hexadecane as the sole carbon source, two glycolipid compounds were detected, the glycolipids share the same saccharide moiety, but the fatty acid moiety were different, one composed of palmitic acid, and the other composed of palmitic acid, myristic acid, octadecanoic acid, and two unsaturated fatty acids (Wang et al., 2013). In *n*-tetracosane culture, only one glycolipid was found, the fatty acid portion of the glycolipid was identified as a mixture of lauric acid, myristic acid, pentadecanoic acid, palmitic acid, octadecanoic acid, and *n*-nonadecanoic acid (Wang et al., 2013). However, in the case of strain As-13-3, when use *n*-hexadecane as the sole carbon source, only one glycolipid was found, and it was identified as Rha-Rha-C_10_-C_10_. We did not find these RL variants in strain As-13-3. Therefore, further investigations are required to identify the other biosurfactant in strain As-13-3, when use different alkanes as the sole carbon source.

### Biosurfactants application outlook in strain As-13-3

In the context of the interest in finding non-pathogenic RL-producing strains rather than pathogenic strains (e.g., *P. aeruginosa*) for commercial production purposes. Thus, strain As-13-3 for non-pathogenic RL-producing strain is more suitable for the industrially-safe production of RLs.

In commercial application of rhamnolipid, the main problem is high cost. There is a great need to develop an efficient rhamnolipid-producing strain and a low cost-effective processing technique. Considering the fact that strain As-13-3 has the advantage of growing fast (within 4 days), and capable of using waste oil as carbon source. Thus, it is promising in application in both bioremediation of oil contaminated environment and industrial production of RLs.

## Conclusions

In this report, *D. maris* As-13-3 was confirmed as biosurfactant-producing strain with alkanes. When using *n*-hexadecane as the sole carbon source, the biosurfactant produced was identified as di-rhamnolipid. The molecular structure of the di-rhamnolipid was characterized as Rha-Rha-C_10_-C_10_, which exhibited satisfying performance of surface activity and emulsification activity. Several genes that played important roles in the process of rhamnolipid biosynthesis were identified including *alkB*, *cyp153*, *algC*, *rmlA*, *rmlB*, *rmlC*, *rmlD*, *rhlA*, *rhlB*, and *rhlC* etc. Further, a complete picture of the di-rhamnolipid synthesis process in strain As-13-3 is shown, which helps in future application in industrial production of RLs.

### Conflict of interest statement

The authors declare that the research was conducted in the absence of any commercial or financial relationships that could be construed as a potential conflict of interest.
