# Supplementary material for: Oil degradation and biosurfactant production by the deep sea bacterium Dietzia maris As-13-3
Source: Front Microbiol. 2014 Dec 16;5:711. doi: 10.3389/fmicb.2014.00711 (PMC4267283; doi:10.3389/fmicb.2014.00711)
Supplement: Supplementary file 1 [file Presentation1.PDF]

Oil degradation and biosurfactant production by the deep sea bacterium *Dietzia maris* As-13-3

Wanpeng Wang[¶], Bobo Cai [¶, #] and Zongze Shao[¶, *]

[¶]Key Laboratory of Marine Biogenetic Resources; State Key Laboratory Breeding Base of Marine Genetic Resources; Collaborative Innovation Center of Deep Sea Biology; Key Laboratory of Marine Genetic Resources of Fujian Province; Fujian Collaborative Innovation Center for Exploitation and Utilization of Marine Biological Resources, Xiamen 361005, China;

[#]Life Science College, Xiamen University, Xiamen 361005, China.

Running title: Biosurfactant characterization from *D. maris*

Wanpeng Wang and Bobo Cai contributed equally to this work.*Corresponding author: Zongze Shao; Mailing address: The Third Institute of Oceanography. Daxue Road178, Xiamen 361005, Fujian, China.

Tel: (+86)592-2195321; Fax: (+86)592-2085376

E-mail: shaozz@163.com;

Table S1 Oligonucleotidesused in this study

Oligonucleotides	Sequence (5′–3′)
alkBf	ATCAACACCGCCCACGAGC
alkBr	GGAGGAAGGCCCAGAACGA
tetRf	ACAGCACCTTCGGCAACC
tetRr	GGCTCGACAGGAGGAACCC
fdf	GAAACCGCACCTGCTACTACCT
fdr	GGCGACTTGGCCGAGAAT
Cyp153f	ACGACACGACCCGCAACT
*Cyp153*r	ACCCTTACGGATGAACTGACC
*fdR*f	GGAGGAGATGCTGTCGATGAA
*fdR*r	CAGGCAACTGCACGGTCAA
addHf	GCAAGAGCGCCAACATCGTC
addHr	CGCCTGGGTGAACTCGTCGTA
acdHf	CTGATTCTCGTGGGCACCG
acdHr	GATGTCGTACTGCGGGTTGG
acSf	CGTGGACGGGGAGAACCTG
acSr	GTTGGGCGGCGAGGAAAT
algCf	TCTACAACCTCATCACCTCCCG
algCr	TCGGCCATCTGCGCCTTGA
rmlAf	ATCAACTGCTCGGCCTCGTC
rmlAr	TGGGTACTCCTCGGTGGTGC
rmlBf	CGAGTCGGTGGCGTTGATC
rmlBr	CGGGTCCTCTGGCTTCTCCT
rmlCf	GCCCGGTCAGGCCAAGTA
rmlCr	TAGATCGCCCGCTGCTCC
rmlDf	ACAACCCGTCGAGCCCGTAT
rmlDr	TTCGAGCAGTTGCTGATGGTG
accD1f	GGGCGTTCCACTGGTGTTGC
accD1r	GGTGCCCTGGCCGAGTATCA
accD2f	CGGGTTACGGTTTCCTGTCC
accD2r	CCTCTGCCATGATCCGTTTG
accA1f	CACCAGAAGGTCATCGAGGAGG
accA1r	CCTGGAGGCGGGTGTTCAT
accBf	GTTCGCGTCCAACATCATCAC
accBr	AGGCGTCGCACAGCATCA
accA2f	ACCTGGGACCTGCGGATGCT
accA2r	CGGGCAGTGCGTGTTCCATC
fabDf	CGAGGCCGATGTCCTGCTGT
fabDr	GGAGGCCATGTGGTGGGTGT
fabBf	GCCGTGCAGGTGTCGAAGGT
fabBr	TTGCCGGTCTGCTCGATGT
fabG1f	CTCGCTCGGCAAGGAGATGA

fabG1r	GGATGAACCGCAGGGTGGACT
fabG2f	ATCGCCATCCACCTCAAGGG
fabG2r	TTGGCTGCGGAGTAGTTGGTCT
fabG3f	GGTTCGCCGAGGCGCTGTAT
fabG3r	AGGGTCTGCCCGGTCATCCA
rhlAf	ACCGACACGAGGAACGACG
rhlAr	GCGAGCGGATGAACACGAT
rhlBf	GGTTCGACGAGCGGTATTTCA
rhlBr	ACCCTGGTCGTGGTGGATGA
rhlCf	TGGCCCTGGTCGTGGTGGAT
rhlCr	TTGTCCGGGTCGTGCCTGCT

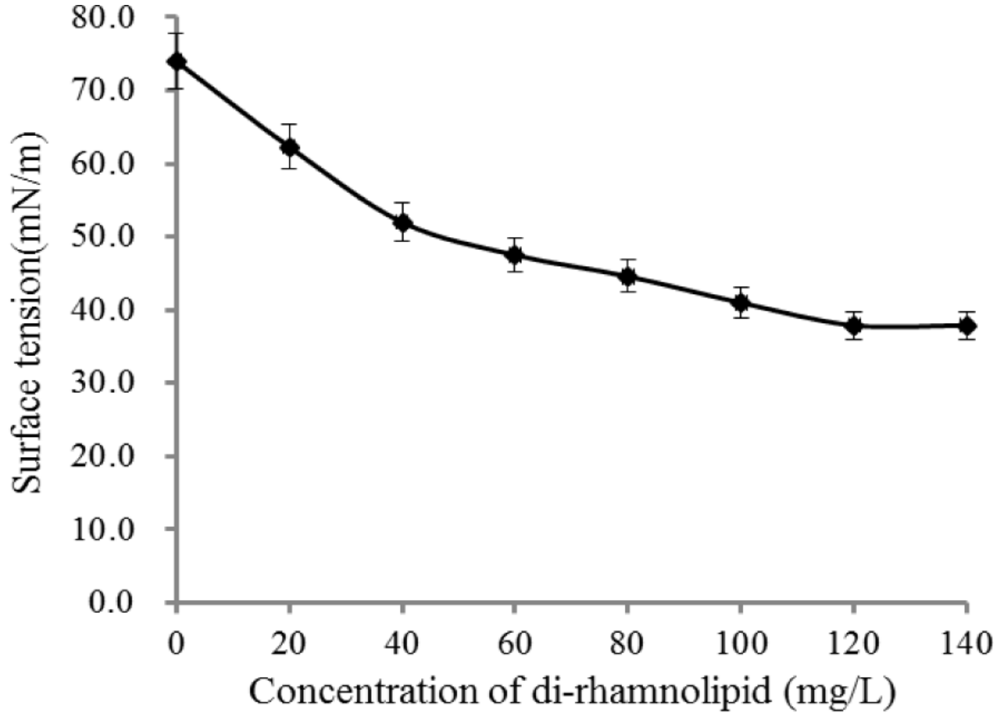

Fig.S1 Determination of CMC value the di-rhamnolipid produced by strain *D.maris* AS-13-3
